# Simulation and Modeling of Optical Properties of U, Th, Pb, and Co Nanoparticles of Interest to Nuclear Security Using Finite Element Analysis

**DOI:** 10.3390/nano12101710

**Published:** 2022-05-17

**Authors:** Elham Gharibshahi, Miltos Alamaniotis

**Affiliations:** 1 Department of Physics and Astronomy, The University of Texas at San Antonio (UTSA), One UTSA Circle, San Antonio, TX 78249, USA; 2 Department of Electrical and Computer Engineering, The University of Texas at San Antonio (UTSA), One UTSA Circle, San Antonio, TX 78249, USA; miltos.alamaniotis@utsa.edu

**Keywords:** uranium (U), lead (Pb), cobalt (Co), and thorium (Th) nuclear materials, nanoparticles, COMSOL simulation, optical properties and band gap energy

## Abstract

In this work, the optical characteristics of uranium (U), lead (Pb), cobalt (Co), and thorium (Th) nanoparticles are fashioned and simulated employing the finite element analysis (FEA) approach concerning multiple particle sizes. Applying finite element analysis, it was found that the simulated absorption peaks of electronic excitations of nuclear nanoparticles are red-shifted from 365 nm to 555 nm for U; from 355 nm to 550 nm for Pb; from 415 nm to 610 nm for Co; and from 350 nm to 540 nm for Th, comparing expanding particle sizes from 60 nm to 100 nm (except for Co, which varied from 70 nm to 100 nm). The FEA-simulated optical band gap energies and far-field radiation patterns were also obtained for nuclear materials. The simulation approach in this research enables the prediction of optical properties and design of nuclear materials before manufacture for nuclear security applications.

## 1. Introduction

Nuclear nanomaterials have attracted much attention due to their rich and unique physical phenomena, as well as electronic and optical properties. The technological importance of nuclear materials largely arises from their utilization in nuclear reactor fuel manufacturing, reactor performance, and long-term storage forms, in terms of both consumed fuels and excess weapons materials.

Among nuclear materials, uranium (U), lead (Pb), cobalt (Co), and thorium (Th) are essential radioactive materials for their applications. For instance, uranium dioxide (UO_2_) is the principal fuel in most significant commercial light-water nuclear power reactors [1,2,3]. Moreover, lead is extensively used in fission reactors as a substitute for sodium, and in the blanket of fusion reactors as a substitute for lithium [4]. Additionally, thorium is used in various nuclear reactors, including light-water, fast spectrum sodium, molten salt, and high-temperature gas-cooled, as well as in fission-fusion hybrid systems and advanced accelerator-driven systems [5,6,7]. The utilization of thorium as a fuel material in the United States continues into presently functioning light-water reactors (LWRs) [5,8]. Furthermore, cobalt is used inside nuclear reactors, for introducing low-level impurity in production substances, and at a significant level within hard-facing composites [9].

The optical characteristics that nanoparticles possess have attracted researchers’ curiosities, owing to their strong extinction efficiency in the visible spectrum [10]. However, the optical properties of nuclear nanoparticles, for both experimental and theoretical purposes, have been studied very scarcely [11,12]. The optical absorption of nuclear nanoparticles is dominated by their localized surface plasmon resonances (LSPR) associated with the collective coherent oscillation of conduction electrons in resonance, accompanied by the incident electromagnetic wave, which relies upon the dielectric constants of the nuclear particles as well as the medium nearby [13,14,15,16,17,18,19].

In this study, we conducted modeling and simulation of the optical characteristics of nuclear nanoparticles containing U, Pb, Co, and Th nanoparticles of different sizes, assisted by the finite element method (FEM) and employing COMSOL Multiphysics.

The finite element method (FEM) is a numerical technique used to perform finite element analysis (FEA) of a physical phenomenon.

The finite element method has several advantages for the simulation of nuclear materials. It possesses the benefit of formulating processes for the basis functions of various orders. Higher orders for the basis functions provide higher-order but exact approaches, which have the significant advantage of improving the precision for a delivered mesh.

Moreover, another benefit of employing the finite element method is that it presents great freedom in the preference of discretization, both in the elements that may be used to discretize space and the basis functions. An additional advantage of FEM is that its theory is well advanced in various fields and applications.

The finite element method’s success in multi-physics analysis is due to its being a very general procedure: solving the resulting equation systems is identical or extremely similar to prominent and efficient processes utilized for structural and electromagnetics analysis. Another reason for the technique’s success is that it makes it easy to increase the order of the elements so that the physics fields can be approximated very accurately.

## 2. Design, Modeling, and Simulation

During the study, the design, modeling, and simulation regarding optical characteristics of nuclear materials containing U, Pb, Co, and Th nanoparticles were conducted using the commercially available finite element analysis software package COMSOL Multiphysics (COMSOL, Inc., Burlington, MA, USA) version 5.4. The specific module ‘Wave Optics’ allows one to study electromagnetic wave propagation and resonance effects in optical applications, analyzing electromagnetic field distributions, transmission and reflections coefficients, and power dissipation in a system of interest.

### 2.1. Simulation of Optical Characteristics of Uranium (U), Lead (Pb), Cobalt (Co), and Thorium (Th) Nanoparticles via COMSOL Multiphysics Software

#### 2.1.1. Theoretical Framework

Mie’s theory [15] is a functional method for defining the optical characteristics of nanomaterials or other spherical particles of any size. This theory provides the basis for predicting the far-field optical properties of nanoparticles [20,21].

Mie’s theory furnishes comprehensive scientific solutions of Maxwell’s equations, intended for the scattering of electromagnetic radiation on particles, including spherical symmetry [22,23]. In this method, the harmonically oscillating electromagnetic fields are represented in terms of a set of spherical vector basis functions so that every term in the expansion describes one of the resonances. The initial expression in the expansion denotes the dipole term, being demonstrated by the quasi-static similarity.

The resolution to Mie’s approach leads to the subsequent total representations regarding the scattering, extinction, and absorption of every particle dimension by contemplating all the numerous oscillations [24,25,26,27]. The cross-sections for the different processes can be described as follows:

(1) $$\Pi_{ext}=\frac{2}{x^{2}}\overset{\infty }{\underset{n=1}{\sum }}\left(2n+1\right)\left[Re\left(c_{n}+d_{n}\right)\right]$$

(2) $$\Pi_{sca}=\frac{2}{x^{2}}\overset{\infty }{\underset{n=1}{\sum }}\left(2n+1\right)\;\left({\left|c_{n}\right|}^{2}+{\left|d_{n}\right|}^{2}\right)$$

(3) $$\Pi_{sca}=\Pi_{ext}-\Pi_{abs}$$

where $\Pi_{abs}$, $\Pi_{ext}$, and $\Pi_{sca}$ denote the absorption, extinction, and scattering cross-sections, respectively. Parameter x denotes

(4) $$x=\frac{2\pi \Omega \varepsilon_{m}}{\Lambda }$$

where Λ expresses the wavelength of the incident beam,$\;\varepsilon_{m}$ indicates the refractive index of the medium, Ω signifies the radius from the particle, and$\;c_{n}$ and $d_{n}$ denote the Mie scattering coefficients of the Riccati–Bessel terms presented below:

(5) $$c_{n}=\frac{j\psi_{n}\left(jx\right)\overset{´}{\psi_{n}}\left(x\right)-\psi_{n}\left(x\right)\overset{´}{\psi_{n}}\left(jx\right)}{j\psi_{n}\left(jx\right)\overset{´}{\xi_{n}}\left(x\right)-\xi_{n}\left(x\right)\overset{´}{\psi_{n}}\left(jx\right)}$$

(6) $$d_{n}=\frac{\psi_{n}\left(jx\right)\overset{´}{\psi_{n}}\left(x\right)-j\psi_{n}\left(x\right)\overset{´}{\psi_{n}}\left(jx\right)}{\psi_{n}\left(jx\right)\overset{´}{\xi_{n}}\left(x\right)-j\xi_{n}\left(x\right)\overset{´}{\psi_{n}}\left(jx\right)}$$

where $\psi_{n}\left(x\right)$ and $\xi_{n}\left(x\right)$ are the Riccati–Bessel functions, and $j=\varepsilon /\varepsilon_{m}$, where $\varepsilon_{m}$ signifies the real dielectric constant of the surrounding medium, and ε indicates the complex dielectric constant of the nanoparticle.

The scattering procedure (Equations (1)–(6)) leads to the term of the extinction cross-section $\Pi_{ext}\;$, as follows:

(7) Πext=24π2Ω3εm23λε2ε1+2εm2+ε22

Here, $\varepsilon_{2}$ and $\varepsilon_{1}$ are the imaginary and real parts of the dielectric constants.

In this study, the analytical model founded on an approximate Mie’s theory has been developed using COMSOL Multiphysics software, whose computations are performed accompanied by the finite element approach.

#### 2.1.2. Simulation Method

The COMSOL Multiphysics package utilizes the finite element method to solve the underlying partial differential equations at hand. It should be noted that the finite element method operates by discretizing the modeling domains into smaller, simpler domains, named elements. The solution is computed by assembling and solving a set of equations over all of the elements of the model. The simulation domain contains a nanoparticle including uranium, lead, cobalt, and thorium in the presence of an air domain trimmed through a *Perfectly Matched Layer* (*PML*). The *Perfectly Matched Layer* (*PML*) is a domain that is added to a model to mimic an open and nonreflecting infinite domain. It sets up a perfectly absorbing domain as an alternative to nonreflecting boundary conditions.

**Figure 1:**
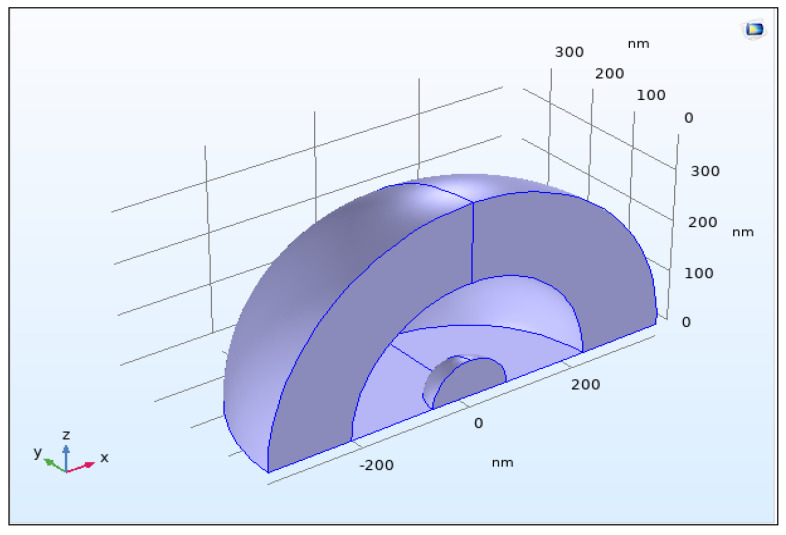
Model geometry of uranium nanoparticle of 40 nm size.

Furthermore, Maxwell’s equations, intended for the scattering of electromagnetic radiation with spherical particles, are solved in the model. The model geometry of a typical uranium nanoparticle of 40 nm size is illustrated in Figure 1.

In this model, the wavelength-dependent optical characteristics, including the absorption cross-section and scattering cross-section, have been simulated for uranium, lead, cobalt, and thorium nanoparticles. The far-field calculations are implemented on the interior boundary of the *PML* for the aforementioned nanoparticles.

Throughout the procedure, the system’s symmetry is preserved via simulating half of the system for nuclear nanoparticles. The simulation computes the local electromagnetic field on each mesh point accompanied by an incident plane wave propagating in the x-direction by the electric field polarized along the z-axis.

The interface applied in the simulation of radioactive nanoparticles was *Electromagnetic Waves, Frequency Domain (emw)*, under the module Wave Optics in COMSOL. The analysis was accomplished in 3D under the frequency domain. This type of approach is appropriate considering that all material properties are constant with respect to field strength, and that the fields change sinusoidally in time at a known frequency range. This is the case of wave-like field solutions of resonant or radiating structures.

**Figure 2:**
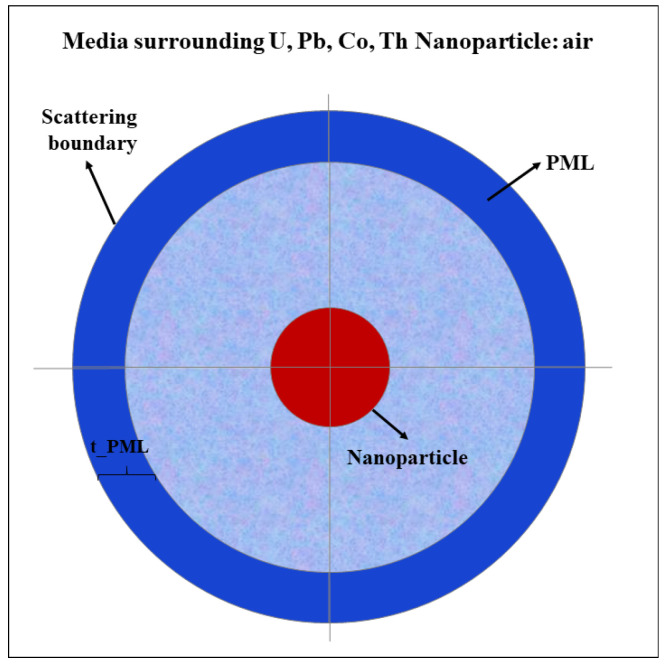
Schematic diagram illustrating the construction of the model for U, Pb, Co, and Th nanoparticle (layers are the thickness of PML, thickness of the air, and sphere radius).

Subsequently, the models of uranium, lead, cobalt, and thorium were created, and nuclear materials were implemented in suitable domains. The *Scattering Boundary Condition* and *Perfectly Matched Layer* (*PML*) were determined, as schematically demonstrated in Figure 2.

**Figure 3:**
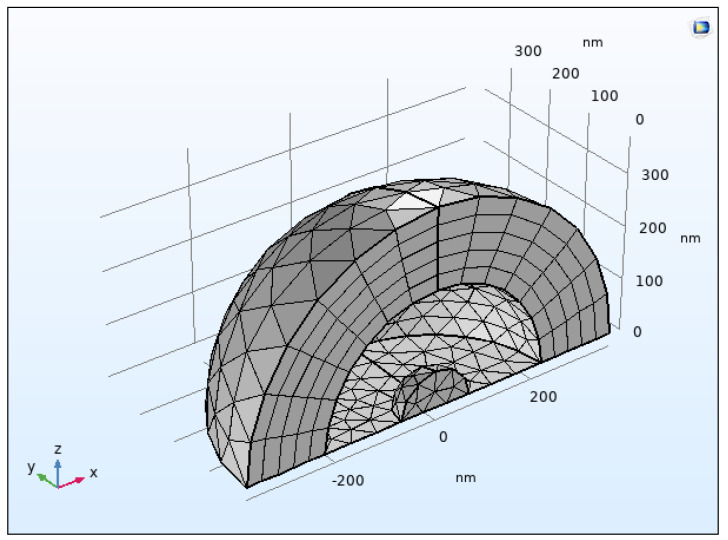
Screenshot of 3D meshing creation of uranium nanoparticle of 40 nm size from COMSOL.

The *Scattering Boundary Condition* is a boundary with the outside world that is transparent to all outgoing waves in the limit that is infinitely far away. The *Perfectly Matched Layer* (*PML*) is a domain that is added to a model to mimic an open and nonreflecting infinite domain. It sets up a perfectly absorbing domain as an alternative to nonreflecting boundary conditions. *Mesh* was executed in the *PML* domain. The remaining structure was designed in a *Free Tetrahedral* fashion (Figure 3).

The *Scattering Boundary Condition* was determined at the exterior boundary of the *PML*. The “*Wave* *Equation, Electric*” physics are supplemented under the “*Electromagnetic* *Waves, Frequency Domain (emw)*” interface. Following the research, *Parametric Sweep* was introduced from 300 nm to 800 nm, with a step size of 10 nm.

In this research, mesh quality is determined by the quality measure, which is skewness. The quality of the mesh guarantees the best analysis results for the problem and minimizes the need for additional analysis runs. Moreover, the sensitivity analysis is a built-in feature in COMSOL Multiphysics, so it does not need to perturb the parameters.

## 3. Results and Discussion

### 3.1. Optical Characteristics

**Figure 4:**
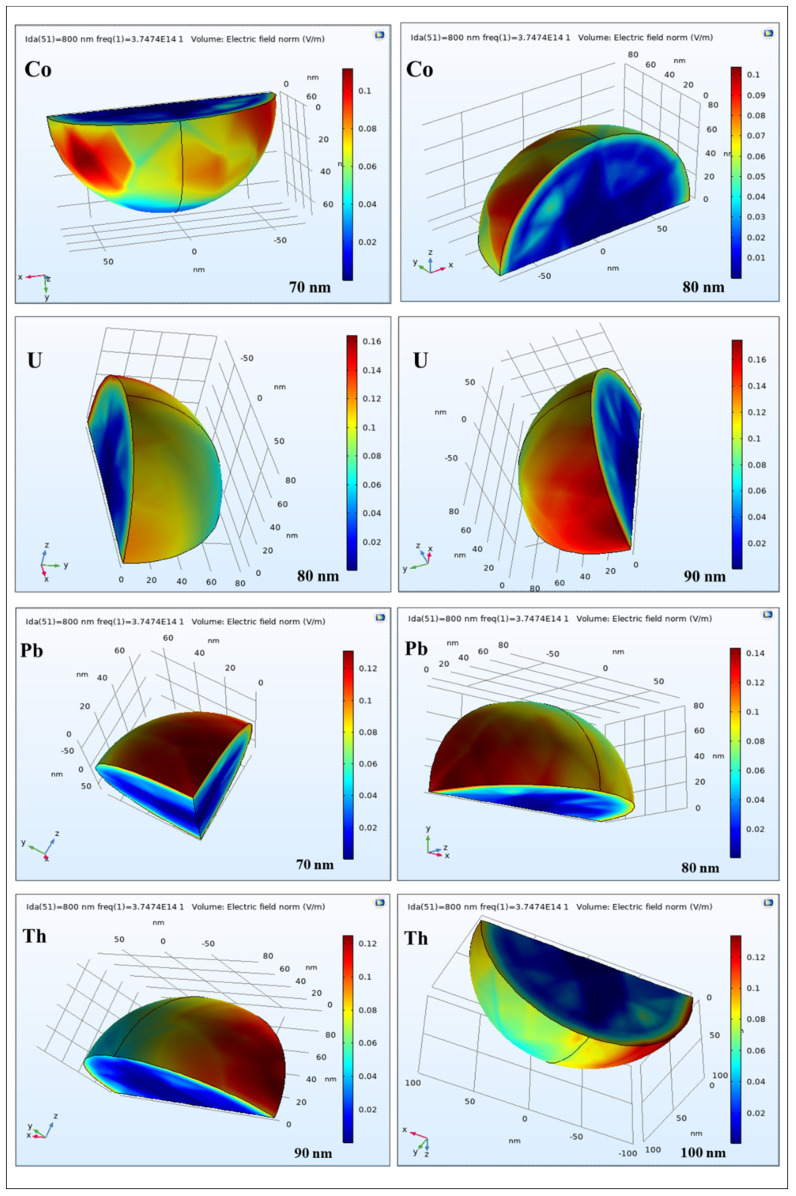
3D plots of U, Pb, Co, and Th nanoparticles from diverse sizes produced via COMSOL, where the color scale illustrates the amplitude of H-field or E-field.

The nanoparticles have been modeled in various sizes, employing the available software. The 3D plots of U, Pb, Co, and Th nanoparticles of diverse sizes acquired from COMSOL are depicted in Figure 4. These are the final results of the finite element analysis.

**Figure 5:**
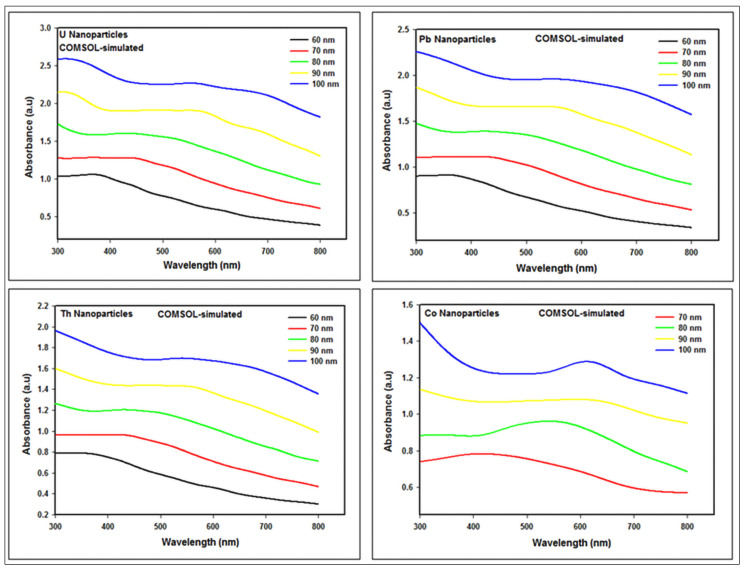
COMSOL-simulated absorption spectra of U, Pb, Co, and Th nanoparticles.

The simulated absorption spectra of U, Pb, Co, and Th nanoparticles of diverse sizes obtained with COMSOL are given in Figure 5, respectively, while simulated maximum absorption peaks of U, Pb, Co, and Th nanoparticles for different particle sizes have been indicated in Table 1.

The outcomes reveal that the simulated absorption peak of the elements is red-shifted depending on particle size from 365 nm to 555 nm for U; 355 nm to 550 nm for Pb; 415 nm to 610 nm for Co; and 350 nm to 540 nm for Th employing COMSOL by increasing particle size from 60 nm to 100 nm, except for Co, which varied from 70 nm to 100 nm.

At this point, it should be noted that the size of Co nanoparticles is limited as a result of the surface scattering effect.

#### 3.1.1. Far-Field Radiation Patterns

The far-field radiation pattern is given by evaluating the squared norm of the far-field on a sphere centered at the origin. Each coordinate on the surface of the sphere represents an angular direction.

The far-field is of interest because it defines how the radiation pattern will behave at different distances from the radiating source. The far-field is defined as the region where the electric and magnetic fields are orthogonal to each other.

The far-field patterns show that a nuclear nanosphere scatters light in the direction of propagation of the incident light (plane wave). The forward–backward asymmetry in the angular distribution of scattered radiation from the nuclear nanoparticles is a consequence of varying particle size.

**Figure 6:**
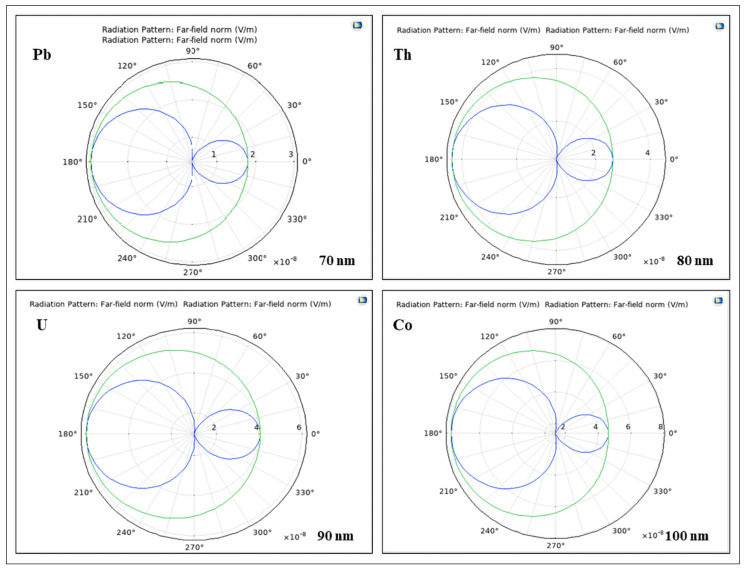
The far-field radiation patterns of U, Pb, Co, and Th nanoparticles of diverse sizes in the E-plane (blue) and H-plane (green) when the wavelength is 800 nm.

Going further, the far-field radiation patterns of uranium, lead, cobalt, and thorium nanoparticles of various sizes for a wavelength of 800 nm are depicted in Figure 6. The H-plane represents the plane containing the magnetic field and the direction of maximum radiation, whereas the E-plane denotes the plane comprising the electric field polarization and the direction of maximum radiation. In this case, the H-plane denotes the *x-y* plane, and the E-plane indicates the *x-z* plane.

#### 3.1.2. Band Gap Calculations

The optical band gap energies can be evaluated applying the primary absorption equation, delineating electron excitation of the valence band to the conduction band, as follows [28,29]:

(8) $$\alpha h\nu =A{\left(h\nu -E_{g}\right)}^{n}$$

where *E_g_* signifies the band gap energy, *α* denotes the absorption coefficient, A is a constant, and *hν* is the photon energy. The parameter n indicates the nature of the optical transition, and it can only receive the values of 1/2, 2, 3/2, or 3, indicating allowed direct, allowed indirect, forbidden direct, and forbidden indirect transitions, respectively.

Tauc’s method demonstrates that considering an allowed direct transition (i.e., *n* = ½), and plotting *(*α*hν)^2^* versus photon energy, the linear part of a similar curve can be extrapolated into the energy axis [30,31,32], including the intersection, which illustrates the appraised value of the band gap energy.

Alternatively, Equation (8) can be refashioned as:

(9) $$\frac{d\left(ln\left(\alpha h\nu \right)\right)}{d\left(h\nu \right)}=\frac{n}{\left(h\nu -E_{g}\right)}$$

**Figure 7:**
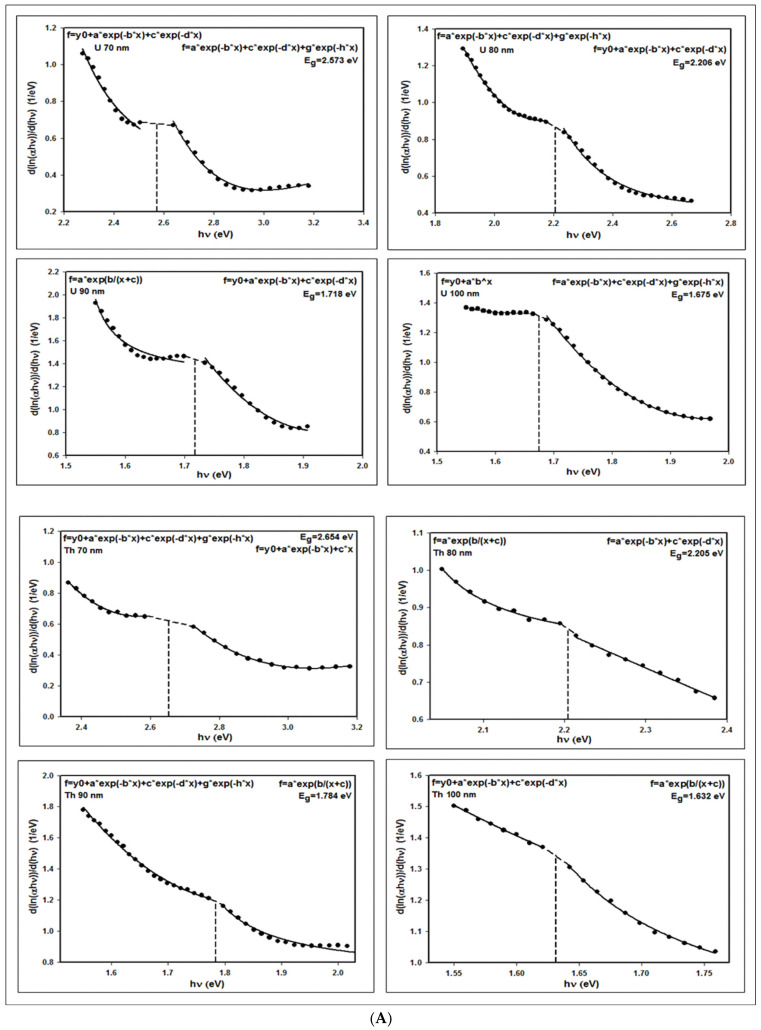

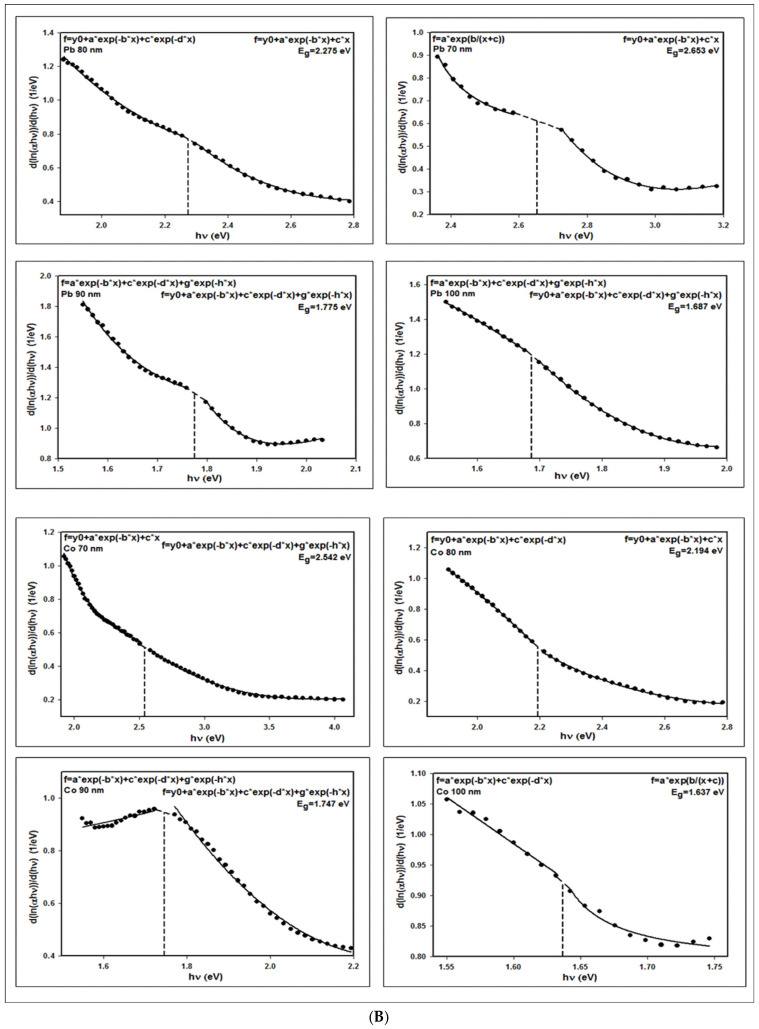
(**A**) COMSOL-simulated plots of $d\left(ln\left(\alpha h\nu \right)\right)/d\left(h\nu \right)$ versus hν for the U and Th nanoparticles, displaying a discontinuity on the computed band gap energy value. (**B**) COMSOL-simulated plots of $d\left(ln\left(\alpha h\nu \right)\right)/d\left(h\nu \right)$ versus hν for the Pb and Co nanoparticles, displaying a discontinuity on the computed band gap energy value.

Apparently, the plot from *d[ln(αhν)]/d(hν)* versus hν diverges at *hν* *= E_g_*; in addition, once more, band gap energy can be evaluated from the plotted discontinuity, as exhibited in Figure 7A,B.

The extracted band gap values of the uranium, lead, cobalt, and thorium nanoparticles employing the aforementioned method are presented in Table 2.

#### 3.1.3. Comparison of the Band Gap of U, Pb, Co, and Th Nanoparticles

**Figure 8:**
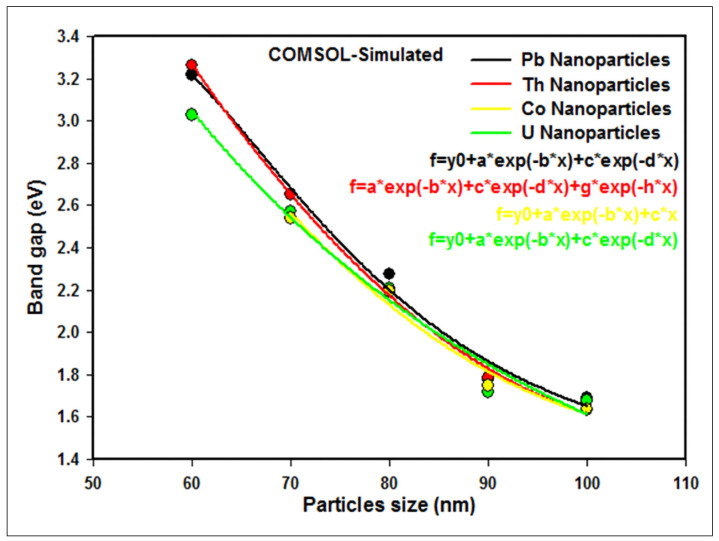
Comparison of the band gap of U, Pb, Co, and Th nanoparticles at different particle sizes.

A comparison between the COMSOL-simulated band gap of U, Pb, Co, and Th nanoparticles of various sizes is provided in Figure 8, based on the values in Table 2.

The nonlinear regression of band gap data diminishes with increasing particle size due to the quantum confinement effect. The results show that the COMSOL-simulated band gap of nanoparticles decreases as follows when increasing particle size from 60 nm to 100 nm (except for Co, which varied from 70 nm to 100 nm): U, from 3.027 eV to 1.675 eV; Pb, from 3.220 eV to 1.687 eV; Th, from 3.263 eV to 1.632 eV; and Co, from 2.542 eV to 1.637 eV. Abnormal results acquired from employing COMSOL Multiphysics software for U, Pb, and Th nanoparticles smaller than 60 nm, and for Co nanoparticles smaller than 70 nm, are due to the surface scattering effect in nanoparticles, and this emphasizes the applicability of the FEA model.

## 4. Conclusions

The COMSOL Multiphysics package and the finite element method have been utilized to define the absorption spectra of U, Pb, Co, and Th nanoparticles. For the aforementioned nuclear nanoparticles 60 nm or greater in size, it is discovered that the COMSOL simulation outcome forecasts the absorption peak maxima well. For instance, the absorption peak for U is red-shifted from 365 nm to 555 nm, for Pb from 355 nm to 550 nm, and for Th from 350 nm to 540 nm with increasing particle size from 60 nm to 100 nm. The theoretical absorption spectra of U, Pb, Co, and Th nanoparticles were further applied to derive the band gap energies. Moreover, the finite element method has several advantages for the simulation of nuclear materials, including formulating processes for the basis functions of various orders, presenting great freedom and a well-advanced theory. This research has substantiated the usefulness of COMSOL for different nuclear nanoparticles investigations. It is predicted that the procedures delineated here will enable design engineers to tailor U, Pb, Co, and Th nanoparticles intended for diverse prospective implementations, including nuclear reactor fuels, reactor operation, weapons materials, and nuclear security.

## Figures and Tables

**Table 1:** The COMSOL-simulated maximum absorption peak of U, Pb, Co, and Th nanoparticles for different particle sizes.

Particle Size (nm)	U		Pb		Th		Co	
	Absorption Peak (nm)	Absorbance (a.u)	Absorption Peak (nm)	Absorbance (a.u)	Absorption Peak (nm)	Absorbance (a.u)	Absorption Peak (nm)	Absorbance (a.u)
60	365	1.0573	355	0.9127	350	0.7876	-	-
70	370	1.2807	365	1.1106	365	0.9641	415	0.7829
80	430	1.6038	425	1.3891	430	1.2036	545	0.9608
90	550	1.9059	540	1.6574	485	1.4375	595	1.0805
100	555	2.2670	550	1.9584	540	1.6978	610	1.2878

**Table 2:** COMSOL-simulated band gap values of *d(ln(αhν))/d(hν)* versus *hν* for the U, Pb, Co, and Th nanoparticles.

Particle Size (nm)	COMSOL-Simulated Band Gap (eV)			
	U	Pb	Th	Co
60	3.027	3.220	3.263	-
70	2.573	2.653	2.654	2.542
80	2.206	2.275	2.205	2.194
90	1.718	1.775	1.784	1.747
100	1.675	1.687	1.632	1.637

## Data Availability

The data are contained within the article.
